# Interpersonal Conflicts in the Unit Impact the Service Quality Rated by Customers: The Mediating Role of Work-Unit Well-Being

**DOI:** 10.3390/ijerph18158137

**Published:** 2021-07-31

**Authors:** Miriam Benitez, Jose M. Leon-Perez, Alejandro Orgambídez, Francisco J. Medina

**Affiliations:** 1 Department of Social Psychology, Faculty of Psychology, University of Seville, 41018 Seville, Spain; leonperez@us.es (J.M.L.-P.); fjmedina@us.es (F.J.M.); 2 Social Psychology Department, Faculty of Psychology, University of Málaga, 29071 Málaga, Spain; aorgambidez@uma.es

**Keywords:** interpersonal conflict, burnout, job satisfaction, service quality, work-unit performance, tourism and hospitality, occupational health and well-being

## Abstract

Social dynamics at work are crucial for understanding how internal processes in an organization are related to their performance and productivity. Following the Service-Profit Chain (SPC) theory, this study analyses, at the work-unit level, how interpersonal conflicts are related to service quality in the hospitality and tourism industry through the shared experience of well-being in the work unit. In other words, we examine the mediating role of two main aspects of work-related well-being in the unit (job satisfaction and burnout) on the relationship between interpersonal conflicts in the unit and customers’ perceptions of service quality. To do so, we conducted a cross-sectional survey study that collected data from 398 service employees (91 work units) and 1233 customers from three and four-star hotels with restaurant in Spain. Using path analysis in Structural Equation Models, our results supported a full mediation model at the work-unit level: interpersonal conflicts in the work unit are related to customers’ service quality perceptions through the work-unit’s well-being (job satisfaction and burnout). Therefore, our findings extend the SPC theory by integrating group dynamics and employees’ experiences, which should be enhanced through occupational health-oriented policies and practices to increase service quality. In this sense, this study has implications for the development of intervention programs aiming at improving the occupational well-being and quality of service in hospitality and tourism settings.

## 1. Introduction

The hospitality industry is a determinant of economic growth, particularly in countries where tourism contributes to an important percentage of the gross domestic product (GDP) [1]. The quality of the service provided by organizations depends to a large extent on the frontline employees of the tourism organization. Providing a high-quality service requires not only having certain skills and competencies, but also experiencing and transmitting positive attitudes and emotions. Indeed, several meta-analyses have shown that employees’ job satisfaction is positively related to customers’ perceived service quality, which is crucial for building long-term relationships with customers and, therefore, for a firm financial performance [2,3,4,5,6,7,8]. Similarly, another meta-analysis comprising 31 studies and more than 10,000 workers concluded that there is a positive relationship between psychological well-being (which incorporates job satisfaction and mental health) and performance, measured in several ways, from leaders’ and peers’ ratings to organizational records [9].

Therefore, it seems that stimulating human resources practices that improve employees’ well-being may repay the investment in these factors through their impact on service quality and job performance. In that sense, recent research in the hospitality and tourism industry (hotels and restaurants) has explored the link between employees’ work-related well-being and service quality [10]. Most of these studies discuss their findings under the lens of the Service-Profit Chain theory (SPC theory: [11,12]), which emphasizes the importance of internal processes (‘support services and policies that enable employees to deliver results to customers’: [11], p. 165) and external service quality (i.e., customer satisfaction and loyalty) for a firm’s financial performance (for a meta-analysis, see [13]).

However, the SPC theory has mainly been applied at the individual level, neglecting the key role of social dynamics at the group or at the organizational level. These internal processes in a company could have an important influence on employees’ well-being and on the quality of the service provided by the organization. In response to this gap, this study links an internal processes in the work unit (interpersonal conflict) to two main aspects of well-being at work (job satisfaction and burnout) analyzing their shared impact on the service quality perceived by customers. Our results may help to move the field forward by incorporating group dynamics as a key elements to improve service quality levels.

We have focused on interpersonal conflicts in the hospitality industry (hotels and restaurants) because they have been considered a work-related stressor with detrimental consequences for employees’ well-being at work. The main reason is that the hospitality profession is a highly competitive sector where service employees have to work in a turbulent business environment of scarce resources, intense pressure, and rapid rates of technological change [14]. Moreover, frontline employees are usually forced to display positive emotional labor characterized by special attention to the customer to offer high-quality service [15]. This stressful context favors the appearance of conflicts among work-unit members that, if they are not managed correctly, could have consequences, both in the well-being of the employee and the service quality provided [16].

### The Service-Profit Chain: Linking Units’ Social Dynamics with Customers’ Appraisals

According to the SPC theory, when employees are satisfied, service quality improves (e.g., [17,18]). In that sense, frontline employees or customer-contact units are the cornerstones of service quality and customer satisfaction and, therefore, they are ultimately responsible for the overall performance of the service organization [19,20,21]. Frontline employees (i.e., employees who deliver the service establishing direct contact with customers) represent the face and the voice of their organizations to customers, and their acts and dynamics determine customers’ experiences [22,23,24]. This is particularly evident in services that require customer presence or participation to provide service such as in hotels and restaurants, making the issue of service delivery more dependent on interpersonal interactions than in other organizations [25].

Moreover, service is often provided by employees working in work units and, therefore, when customers evaluate service quality, it is often assesses the quality of service delivered by the work unit as a whole, rather than service offered by individual work-unit members [20]. Hence, in these work units, members spend a long time working together and interacting with customers as a collective entity, which, in turn, leads to customers to experience similar stimuli and share perceptions of service quality.

In addition, internal social processes in the work unit may determine the interaction between employees and customers, which, in turn, may affect how customers evaluate such encounters. Following this rationale, some authors have suggested that interpersonal conflicts in the unit are negatively linked to service quality [26]. For example, Leon-Perez et al. [27], found a negative relationship between interpersonal conflicts within the work unit and self-reported measures of service quality in a sample of 55 units of a vehicle safety and emissions inspection company. Although the effects of interpersonal conflicts on group effectiveness have been studied extensively, these complex relationships have not been fully established and many inconsistencies persevere [28,29]. One of the reasons could be that previous studies used only employees’ self-reported data; thus, failing to provide insights into the service encounter, which ideally requires data from both employees and their customers. For that reason, we hypothesize that interpersonal conflicts within the unit will be reflected negatively in their service quality as a consensus measure reported by their customers (for a review about consensus composition models, see Klein, Conn, Smith, and Sorra [30].

**Hypothesis** **1.** *At the work-unit level, interpersonal conflicts within the work unit will be negatively related to customers’ shared perceptions of service quality.*

Although interpersonal conflicts in the work unit may directly impact customers’ perceptions of service quality, the SPC theory allows us to suggest a path in which interpersonal conflicts within the unit negatively impact customers’ shared perceptions of service quality by decreasing the employee satisfaction and well-being in the unit (see also Whitman, Van Rooy, and Viswesvaran, [31]). In this regard, interpersonal conflicts at the work-unit level bring about strong unpleasant feelings and strained responses [14,32,33,34]. For example, results from both a cross-sectional and a diary study over a period of two weeks revealed that “conflict may lead to depressive symptoms, which make people even more vulnerable to conflicts, indicating a vicious circle with high psychological and economic costs” ([35] p. 31). 

Additionally, there is evidence suggesting that well-being can also be socially induced and passed on (social contagion: [36,37]). Therefore, considering that shared perceptions of the social processes within a work unit can influence work-related well-being at an aggregated level of analysis, experiencing interpersonal conflicts within the work unit may both decrease job satisfaction and increase burnout of the work unit.

**Hypothesis** **2a.** *At the work-unit level, interpersonal conflicts within the work unit will be negatively related to job satisfaction.*

**Hypothesis** **2b.** *At the work-unit level, interpersonal conflicts within the work unit will be positively related to burnout.*

In addition, well-being at work is considered a key factor for developing a satisfactory relationship between the provider and the customer, particularly in the tourism and hospitality sector [38]. Moreover, this association between work-related well-being and service quality has been tested at the unit level of analysis. As Harter et al. [6] concluded after conducting a meta-analysis that comprised 198,514 employees nested in 7939 work-units from 36 organizations, there is a positive association between job satisfaction and engagement and performance at the work-unit level, which explains both customers’ satisfaction and organizations’ productivity and profitability (e.g., lower levels of turnover and lesser number of work accidents at the organizational level). Equally, previous studies have followed a similar rationale and have analyzed how employees’ well-being at work mediates the relationship between group processes and customer satisfaction [39,40,41]. In particular, the negative side of work-related well-being (burnout, which comprises two core dimensions: emotional exhaustion and cynicism) has been related to a diminished service quality, both at the individual [39] and the group level [41].

**Hypothesis** **3a.** *At the work-unit level, employee satisfaction will mediate the relationship between interpersonal conflicts and customers’ shared perceptions of service quality.*

**Hypothesis** **3b.** *At the work-unit level, employee burnout will mediate the relationship between interpersonal conflicts and customers’ shared perceptions of service quality.*

In sum, following the SPC theory, this study analyzes the mediating role of work units’ satisfaction and burnout (i.e., well-being) on the relationship between interpersonal conflicts in the unit and customers’ perceptions of service quality. To do so, we have incorporated measures from different sources to examine the relationship between internal processes (interpersonal conflicts and well-being in the work unit) and external results (customers’ service quality ratings) [42], and we have considered the work unit or the group of employees with whom a particular set of customers interact as the appropriate unit of analysis in service settings, rather than the individual employee-customer dyad [20,43,44]. To sum up, our findings may have relevant practical implications for organizations, incorporating group dynamics as strategical human resources practices to improve service quality levels.

## 2. Materials and Methods

### 2.1. Study Design and Procedure 

This study followed a cross-sectional survey design, using questionnaires for data collection. To carry out the study, the researchers contacted managers from three- and four-star hotels, with restaurant service, in Andalusia (Spain). Hotel managers were contacted via telephone. After receiving an explanation regarding the aim of the study, they were invited to participate and their permission was requested to administer a questionnaire to a group of their employees and customers. 

Data were collected at the service site (real-time service quality approach). The *real-time service quality approach* is associated with an assessment that occurs during an on-site experience and reflects a direct evaluation of the focal service. In contrast, the post hoc *service quality approach* is associated with an assessment that occurs sometimes after the on-site experience and can also reflect the customer experience with different service providers. As this study focuses on the prediction of customers’ perceptions of service quality about the focal service, and because focal service information is useful for efforts related to management, design, or policy [45], we followed a *real-time service quality approach.*

The questionnaire administration processes took around 20 min for employees and 5 min for customers. Data were gathered over two high-season days. The confidentiality and anonymity of the answers were guaranteed for both samples. Participation was voluntary, with informed consent following the European regulations on personal data management and research ethics according to the Spanish Association of Psychology.

### 2.2. Participants and Inclusion Criteria

Our study included two sources of information: frontline employees and customers. Then, the responses of the employees were aggregated into work units (see data analysis strategy). Although participation was voluntary, we included the following inclusion criteria:

Regarding the inclusion criteria, we focused on frontline employees that had to make contact regularly with customers as part of their daily work. Employees filled in the questionnaire during breaks, at the beginning, or the end of their shifts. In addition, hotel customers filled in the questionnaire when checking out. To be eligible, the customers had to have spent at least one night in the hotel. For restaurants, the researchers requested the cooperation of customers immediately after their consumption experience (lunch or dinner) with the main restaurant. In other words, restaurant customers filled in the questionnaire after the service transaction had been completed (i.e., after paying the check). The researchers were present to help employees and customers in case of difficulties with filling in the questionnaires. Finally, regarding work units, we required a minimum of 3 responses from contact employees per work unit and for which service quality was evaluated by a minimum of 10 customers.

We collected self-report data from 398 service employees (receptionists and waiters) and from 1233 customers from 42 hotels and 42 restaurants. The initial sample consisted of 99 work units; however, after applying our inclusion criteria, usable surveys were obtained from 91 work units (91.1%): 49.7% of the units were receptionists and 50.3% were waiters. Information was gathered from a minimum of 3 and a maximum of 11 members of each work unit, with an average of 4.94 members (*SD* = 3.1). In 57.6% of the sample of work units, three members were surveyed and in 42.4%, more than four members were surveyed.

Regarding participants in the receptionists’ work-units, 44.7% were women and 53.8% men (and 1.5% of respondents whose sex was not specified). Participants had different educational levels: elementary school (9.0%), high school (17.6%), high school graduates (20.6%), university graduates (49.2%), and 2.5% without any level of education. Concerning age, 53.8% was situated in the rank of 18 and 29 years old, 33.2% between 30 and 39 years, 7.5% between 40 and 49 years, leaving 5% over 49 years. Position tenure ranged from a few months to 32.6 years, with a mean age of about 5 years (*SD* = 8.45), and 40.7% of the sample consisted of temporary workers.

Regarding participants in the waiters’ work units, about 56.1% were men. Age ranged in 51.0% between 18 and 29 years old, in 29.3% between 30 and 39 years, in 15.7% between 40 and 49 years, and in 3.6% over 49 years. Waiters had different educational levels: elementary school (27.3%), high school (33.8%), high school graduates (27.3%), university graduates (8.6%), and 3.0% without any level of education. Regarding contract type, 47.0% were temporary workers. 

Finally, the customer sample consisted of 1233 clients from the 91 work units (54% males and 46% females) and the response rate was 95%. Information was gathered from a minimum of ten customers and a maximum of 20 for each of the 91 work units, with an average of about 13 customers.

### 2.3. Instruments and Measures

*Interpersonal Conflicts*. This variable was measured through the interpersonal conflict at work questionnaire (CIT), in its Spanish version [46], that comprised both task-oriented conflicts (e.g., “How often do people in your team disagree about opinions regarding the work being done?”) and relationship-oriented conflicts (e.g., “How much plotting takes place behind the scenes?”). Response anchors ranged from 1 (*none*) to 5 (*to a great extent*). Cronbach’s alpha coefficient was 0.87. The higher the score, the higher the level of interpersonal conflicts experienced.

*Work-unit well-being.* We defined work-related well-being at the unit level in line with Rothmann’s approach [47], who considered that work-related well-being is a multifactorial construct that includes job satisfaction and burnout, among other factors. Therefore, we measured burnout and job satisfaction as key indicators of work-unit well-being. Regarding *Work-unit Burnout,* this factor was measured using the emotional exhaustion dimension (3 items, e.g., “I feel burnout at my work”) and the cynicism dimension (3 items, e.g., “I feel that, in my job, I am too hard on the customers”) from the Spanish version of the Maslach Burnout Inventory (MBI-GS; [48]). Traditionally, burnout involves experiences of exhaustion (emotional exhaustion), distant or negative attitudes and feelings towards the people one is working with (cynicism), and the development of negative attitudes and feelings of incompetence regarding one’s professional role (lack of efficacy) [49]. However, lack of efficacy has been criticized as reflecting a personality characteristic rather than a genuine burnout component [50]. Thus, some research has supported a two-component model, with the inclusion of emotional exhaustion and cynicism dimensions, called the “core of burnout” (e.g., [50,51]). For this reason, in the present study, we considered cynicism and emotional exhaustion as key symptoms of burnout. All the items of the MBI-GS were scored on a five-point frequency rating scale from 1 (*never*) to 5 (*always)*. The consistency coefficient (Cronbach’s alpha) of the scale was 0.80. Higher scores on exhaustion and cynicism were indicative of higher levels of burnout.

In addition, *Work-unit Job satisfaction* was measured with the 5 items from the Spanish version of Hartline and Ferrell’s scale [17]. This scale follows the conception of job satisfaction as the well-being feeling derived from specific work-related facets: overall job, supervisor, organization’s policy, support provided by the organization, and opportunities for advancement with the organization. Response anchors ranged from 1 (*very dissatisfied*) to 7 (*very satisfied*). Cronbach’s alpha coefficient was 0.89. Higher scores indicate higher job satisfaction.

*Customers’ perceptions of service quality*. We used Sánchez-Hernández, Martínez-Tur and Ramos’s questionnaire to measure service quality [52] according to two dimensions: functional (8 items, e.g., “The services in this hotel/restaurant are efficiently provided”) and relational (6 items, e.g., “The employees show a real interest in creating a good relationship with the clients”). All items are scored on a seven-point rating scale ranging from 1 (*strongly disagree*) to 7 (*strongly agree*). The consistency coefficient (Cronbach’s alpha) for the scale was 0.95. Higher scores indicate higher service quality.

### 2.4. Data Analysis

First, we aggregated data to the work-unit level. The conceptual rationale for using an aggregated measure of variables in the study was discussed in the introduction. However, as Klein, Dansereau and Hall [53] showed, aggregation must also be accompanied by statistical justification. We used Intraclass Correlation Coefficient (ICC (1)), and also ADI indexes (AD_*M*_ index) [54] in order to justify aggregation to higher levels of analysis [55].

To test within-unit agreement, we computed an Average Deviation index (AD_*M*_ index) based on the deviation from the item mean for interpersonal conflicts, burnout, job satisfaction, and service quality [55,56]. According to Burke and Dunlap [55], and taking into account the number of response options and their verbal anchors, for interpreting AD values equaled 1 (c/6 = 1) when the response scale is a Likert-type 5-point scale (interpersonal conflicts and burnout) within-unit agreement is acceptable when the values are equal to or less than 1. When the response scale is a Likert-type 7-point scale (job satisfaction and service quality) within-unit agreement is acceptable when the values are equal to or less than 1.16. To determine between-unit differentiation, we computed the interclass correlation coefficient (ICC(1); see [57]).

The mean AD_*M*_ values obtained for the study variables were as follows: interpersonal conflicts = 0.63 (*SD* = 0.22); burnout = 0.67 (*SD* = 0.24); job satisfaction = 0.81 (*SD* = 0.32); service quality = 0.57 (*SD* = 0.25). The ICC(1) obtained for each variable was: interpersonal conflicts = 0.10; burnout = 0.29; job satisfaction = 0.20; service quality = 0.22. As we can observe, ICC(1) values were all above 0.10; thus, supporting the use of a multilevel approach [58]. According to the ICC(1) interpretation, 10% of the interpersonal conflicts variance, 29% of the burnout variance, 20% of the job satisfaction variance, and 22% of the service quality variance may be due to work-unit membership. Based on these results, we concluded that levels of within-unit agreement in the present study were sufficient to aggregate work-unit members’ scores on all variables to the work-unit level.

We also carried out a one-way analysis of variance (ANOVA) to ascertain whether there was statistically significant between-unit discrimination in the study variables [57]. The results showed significant differences for interpersonal conflicts, *F* (90, 295) = 1.5, *p* < 0.01; burnout, *F* (90, 302) = 1.83, *p* < 0.01; job satisfaction, *F* (90, 302) = 1.88, *p* < 0.01; service quality *F* (90, 304) = 1.63, *p* < 0.01. These results suggest an adequate between-unit differentiation in all variables and support the viability of these measures.

The research hypotheses were analyzed using the path analysis method. The analyses were conducted in LISREL 8.54 following the main recommendations proposed in the literature (e.g., [59,60,61]). Missing data were dealt with using mean substitution. Maximum likelihood estimation methods were used, and the input for the analysis was the covariance matrix. As no statistical test or critical values are available to determine the adequacy of absolute indexes, researchers also recommend the computation of relative goodness-of-fit [62,63]. Thus, the following absolute and relative goodness-of-fit indexes were calculated: the chi-square goodness-of-fit statistic (χ^2^), Comparative Fit Index (CFI), Tucker–Lewis Fit Index (TLI), Root Mean Square Error of Approximation (RMSEA), and Standardized Root Mean Square Residual (SRMR).

The mediation analysis was performed using the bootstrapping method. Compared to other methods, bootstrapping is one of the methods with the highest statistical power. This method involves bootstrapping the regression model and calculating the empirical confidence intervals [64]. A total of 10,000 resamples were created for the estimation of the Percentile (PC 95%) confidence intervals of the mediation effects. The mediation effect is significant (*p* < 0.05) when the confidence interval does not include zero (0).

## 3. Results

### 3.1. Descriptive Results

We first provide descriptive results for the work units’ main variables of this study such as means, standard deviations, and Pearson correlations. As it can be seen in Table 1, interpersonal conflicts presented a significant negative relationship with job satisfaction (*r* = −0.30, *p* < 0.01), and a positive relationship with burnout (*r* = 0.30, *p* < 0.01). Additionally, job satisfaction was positively related to service quality (*r* = 0.24, *p* < 0.01) and burnout was negatively related to service quality (*r* = −0.35, *p* < 0.01). However, interpersonal conflicts were not related to service quality (*r* = −0.04, *ns*).

### 3.2. Mediation Analysis

**Figure 1:**
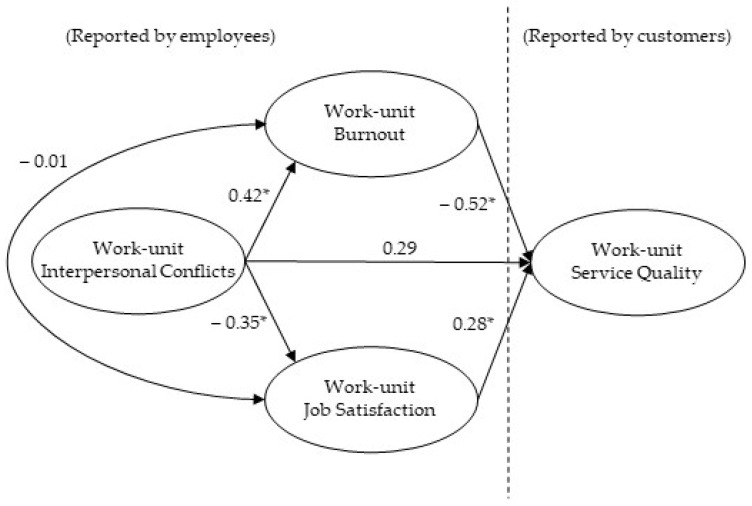
Partial mediation model. * *p* < 0.01. Standardized coefficients.

**Figure 2:**
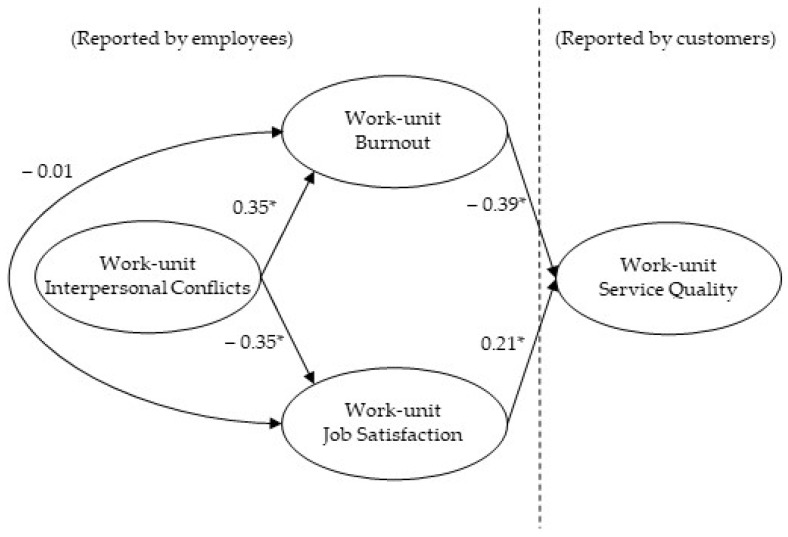
Full mediation model. * *p* < 0.01. Standardized coefficients.

Our proposed model (full mediation, M1) and the alternative model (partial mediation, M2) were compared computing multiple regressions models. The two models showed a good fit: M1 model χ^2^(2) = 1.83, *p* = 0.39, TLI = 1.00, CFI = 1.00, RMSEA = 0.00 (90% CI: 0.00, 0.20), and SRMR = 0.04; M2 model χ^2^(1) = 0.07, *p* = 0.80, CFI = 1.00, TLI = 1.00, RMSEA = 0.00 (90% CI: 0.18), and SRMR = 0.01, with CFI and TLI coefficients higher than 0.95 and RMSEA and SRMR coefficients below 0.08. The path from interpersonal conflicts to service quality in the M2 model was not significant (Beta = 0.29, *ns*, see Figure 1). Given that both models obtained identical indexes, we retained the full mediation model because it presented greater parsimony with a lesser number of connections (see Figure 2).

Regarding the mediation effect of burnout and job satisfaction, burnout was a significant mediator (*p* < 0.05) in the relationship between interpersonal conflicts and service quality (PC 95% CI: −0.24, −0.04). Job satisfaction was also shown as a significant mediator (*p* < 0.05) between interpersonal conflicts and service quality (PC 95% CI: −0.17, −0.01). Considering the total indirect effect of interpersonal conflicts on service quality, 75.75% was mediated by burnout and 24.25% was mediated by job satisfaction (see Table 2).

## 4. Discussion

As the SPC theory proposes, the link between employees’ well-being at work (job satisfaction and burnout) and customers’ service quality perceptions are crucial for organizational sustainability and productivity. Furthermore, group social dynamics such as interpersonal conflicts may determine employees’ satisfaction and burnout, which, in turn, influences customer perceptions of service quality. Our results confirmed this full mediation model at the work-unit level: interpersonal conflicts in the work unit are related to customers’ service quality perceptions through employee well-being at the work-unit level in both its positive (job satisfaction) and negative dimensions (burnout).

These findings have relevant implications for theory and practice. From a theoretical point of view, our results confirm the link, in the service-profit chain, between employees’ well-being states at work (satisfaction and burnout), and customers’ satisfaction (quality of service). Indeed, our results support the idea that employees’ well-being at work has an important impact on customers’ perceived quality of service, which is crucial for organizational profit and growth [12]. According to the SPC theory, employees’ states are primarily derived from organizational policies and practices that support and enable employees to deliver high-quality services to customers. Our results suggest that not only organizational policies and practices are important (e.g., work design, or HR practices) but also social interactions among units’ members and group dynamics should be considered when striving for excellence in service organizations.

Moreover, our results extend the SPC theory by incorporating relevant group dynamics (such as interpersonal conflicts) that determine employees’ shared perceptions of satisfaction and burnout, which, in turn, is associated with the service quality perceived by customers. In that sense, our findings demonstrate that current service organizations are demanding work environments for employees because workers must follow very high-quality standards with a low tolerance to mistakes [14]. In this context, discrepancies between employees may arise and social relationships can be easily deteriorated, causing interpersonal conflicts among unit members that can be detrimental to service quality [64]. These findings are in line with a previous study conducted in 156 bank branches, revealing that interpersonal conflicts deteriorate the affective climate of the work-unit [65]. The logic behind this idea is that social relationships between employees of an organization transfer to the interaction between employees and customers, which ultimately yields the service quality that customers experience [66].

In a similar vein, our findings are consistent with the thesis of *feeling good leads to doing good* [67] and *the happy-productive worker hypothesis* [68,69]. When work-unit members feel happy (satisfied, engaged), they display helping behaviors and cooperation among workers, and are more inclined to express positive emotions at work directed towards customers [18,70]. On the other hand, work-unit burnout reduces the levels of customer service quality [40,71,72]. In the context of our study, two complementary findings may explain our results: (1) when workers experience negative emotions and strain, it is the captured by customers through emotional contagion processes, affecting the customers’ perceived service quality [73]; (2) a study conducted in The Netherlands, that used external observer ratings of service employee–customer interactions, revealed that cynical employees exhibited more negative actions towards their customers; therefore, customers perceived lower service quality [40]. Indeed, the indirect effect of interpersonal conflicts on service quality seems to be higher for burnout than job satisfaction.

In light of these results, from a practical point of view, managers and supervisors should promote a work-unit context with positive experiences, avoiding negative experiences such as tension or anxiety caused by interpersonal conflicts. Indeed, previous studies have highlighted that effectively managing interpersonal conflicts in the wok unit is crucial for performance [29,74]. Findings suggest that customers have a perception of the service quality because of an overall perception of their interactions with employees. For that reason, organizations need to take care of interpersonal conflict and well-being among all the members that interact with customers. Thus, conflict management training may help to create a good service climate in the work unit that allows employees to establish positive social relationships with both colleagues and customers and deliver a good service (see Leon-Perez et al. [27]). Additionally, team development and positive interventions can complement workplace stress management and health promotion interventions to foster satisfaction and well-being in the work unit (for reviews, see [75,76]).

### Limitations and Further Research

This study has also some limitations that should be noted. It is noteworthy the cross-sectional nature of our data which did not permit us to explore these processes over time and to analyze possible reversed causal relation between our main variables. That is, interpersonal conflicts not only trigger poor work-related well-being, but can also be the result of it [77]. Additionally, customer emotions could have a direct impact on employees by reducing job satisfaction and increasing the experience of burnout [68,78,79]. In other words, satisfied employees show better service performance leading to satisfied customers, but also dissatisfied customers exhibit stressful behaviors that cause employee strain and low job satisfaction. In this sense, Ryan, Schmit, and Johnson [80] showed that customer satisfaction is more likely to cause employee job satisfaction than the other way around. These reserved causal relationships clearly need future studies with a longitudinal design.

Finally, the sample of this research only included hotel and restaurant employees’ data and, therefore, results generalization is somewhat limited. Testing the present model on other samples, including different types of actors (employees, supervisors), companies, and sectors, will provide good opportunities to explore the generalization of the proposed model. In doing so, future studies may explore the role of different types of conflicts (see [28]).

## 5. Conclusions

This study emphasizes the role of satisfaction and burnout at the aggregate group or unit level of analysis in the relationship between group dynamics (interpersonal conflicts) and service quality (measured through customers’ ratings), which is central to an understanding of organizational performance in service contexts. Our results extend SPC theory by incorporating group dynamics and employees’ experiences that can be improved through occupational health-oriented policies and practices.

## Figures and Tables

**Table 1:** Work unit means, standard deviations, and bivariate correlations among the main variables of the study (n = 91 work units).

Variable	Mean	SD	1	2	3	4
1. Interpersonal conflicts	2.38	0.61	(0.87)			
2. Job satisfaction	4.89	0.96	−0.30 **	(0.89)		
3. Burnout	2.41	0.26	0.30 **	−0.11	(0.80)	
4. Service quality	5.89	0.30	−0.04	0.24 *	−0.35 **	(0.95)

Note: * *p* < 0.05 two-tailed; ** *p* < 0.01 two-tailed. Cronbach’s alpha coefficients, representing the reliability of the scales at the individual level, are in the correlation matrix diagonal.

**Table 2:** Indirect and total effects of burnout and job satisfaction on service quality at the work-unit levels (n = 91).

Predictor Variable (X)	Mediator Variable (M)	Result Variable (Y)	X → M	M → Y	Direct	Indirect (PC 95% CI)	Total (PC 95% CI)
Interpersonal conflicts	Burnout Job satisfaction	Service quality	0.34 −0.58	−0.37 0.14	— —	−0.125 (−0.24, −0.04) −0.080 (−0.17, −0.01)	−0.125 (−0.24, −0.04) −0.080 (−0.17, −0.01)

Non-standardized coefficients. Percentile Confidence Intervals based on 10,000 resamples (95%).

## Data Availability

The data that support the findings of this study are available on request from the corresponding author, (Benitez, M.). The data are not publicly available due to private agreements with some organizations that participated in the study.
